# Unraveling the dual immunomodulatory and immunogenic roles of the central conserved cysteine-rich region in respiratory syncytial virus G protein

**DOI:** 10.3389/fmicb.2026.1794062

**Published:** 2026-03-11

**Authors:** Juan Gutman, Ana Luz Paletta, Federico Birnberg-Weiss, Cecilia Arahi Prato, Analía Boudgouste, Carla Jimena Goldin, Santiago Sastre, Alana Brooke Byrne, Pablo Pakciarz, Fernando Pedro Polack, Julia Dvorkin, Ari Zeida, Mauricio Tomas Caballero, Verónica Inés Landoni, Gabriela Cristina Fernández, María Virginia Tribulatti, Damian Alvarez-Paggi, Sebastián Andrés Esperante

**Affiliations:** 1 Centro de Rediseño e Ingeniería de Proteínas (CRIP), Consejo Nacional de Investigaciones Científicas y Técnicas (CONICET), Escuela de Bio y Nanotecnologías (EByN), Universidad Nacional de San Martín (UNSAM), Buenos Aires, Argentina; 2 Instituto de Investigaciones Biotecnológicas (IIBio), CONICET, Escuela de Bio y Nanotecnologías (EByN), Universidad Nacional de San Martín (UNSAM), Buenos Aires, Argentina; 3 Laboratorio de Fisiología de los Procesos Inflamatorios, Instituto de Medicina Experimental (IMEX)-CONICET, Academia Nacional de Medicina, Buenos Aires, Argentina; 4 Departamento de Biofísica, Facultad de Medicina, Universidad de la República, Montevideo, Uruguay; 5 Centro de Investigaciones Biomédicas (CEINBIO), Facultad de Medicina, Universidad de la República, Montevideo, Uruguay; 6 Centro Infant de Medicina Traslacional (CIMeT), Escuela de Bio y Nanotecnología, Universidad Nacional de San Martín, Buenos Aires, Argentina; 7 Fundación INFANT, Buenos Aires, Argentina; 8 Departamento de Bioquímica, Facultad de Medicina, Universidad de la República, Montevideo, Uruguay

**Keywords:** host/pathogen interactions, immunomodulation, immunopathogenesis, redox, RSVG central conserved domain

## Abstract

**Background:**

Respiratory syncytial virus (RSV) causes severe respiratory disease in infants and high-risk adults, in part by subverting host immunity. The RSV G glycoprotein’s central conserved cysteine rich domain (CCD) contains a CX3C motif implicated in immune modulation, but the relationship between its redox state, structural conformation, and immune modulatory function remains unclear.

**Methods:**

In this study, we recombinantly expressed a CCD-derived peptide (Gpep, residues 149–196), determined its redox-dependent folding by reversed-phase HPLC (RP-HPLC) and biophysical analyses, and assessed its function using murine dendritic cell and human neutrophil assays alongside pediatric serology.

**Results:**

Kinetic analyses by RP‑HPLC and biophysical techniques showed that reduced Gpep rapidly folds through a predominant intermediate to yield an oxidized monomer; conversely, higher concentrations drive intermolecular disulfide isomerization and covalent oligomer formation. Functionally, Gpep inhibited dendritic cell activation elicited by both LPS- and UV-inactivated RSV. In addition, Gpep suppressed multiple human neutrophil responses, including chemotaxis, CD11b upregulation, reactive oxygen species production, myeloperoxidase release, and NET formation, without inducing cytotoxicity. In contrast, oligomerized Gpep lacked immunosuppressive activity. Serological analysis of an ambulatory pediatric cohort (0–72 months) showed a transient increase in the anti-F/anti-G IgG ratio following early RSV exposures, consistent with preferential maturation of F-directed responses.

**Discussion:**

We propose a redox‑dependent immune‑evasion model in which secreted, monomeric G mediates transient immunosuppression that is halted by disulfide‑driven oligomerization of membrane‑bound G and F proteins. These findings support a mechanistic association between the redox state of RSV G, its oligomeric behavior, and its immunomodulatory properties.

## 1 Introduction

Respiratory syncytial virus (RSV) is a leading cause of severe lower respiratory tract infections, particularly affecting children under 5 years of age and high-risk populations such as the elderly and immunocompromised individuals (Penders et al., 2025; Li et al., 2022; Wildenbeest et al., 2024; Chatzis et al., 2018). Although RSV infection activates the adaptive immune system, the resulting immunity is typically short-lived and non-sterilizing. This leads to recurrent mild RSV infections throughout life and severe disease episodes in vulnerable groups, including immunocompromised individuals and those over 65 years old (Agac et al., 2023). Several viral factors alter the host immune microenvironment, promoting disease pathogenesis and recurrent infections (Parsons et al., 2024; Anderson et al., 2020). Most mechanisms of RSV immune evasion act by suppressing the antiviral interferon response, thereby disrupting both innate and adaptive immunity (Chirkova et al., 2013; Van Royen et al., 2022; Merritt et al., 2023). The RSV genome is a negative-sense, single-stranded RNA encoding 11 proteins from 10 open reading frames (ORFs): NS1, NS2, N, P, M, SH, G, F, M2-1, M2-2, and L (Collins et al., 1996). Among these, the F and G glycoproteins are key surface proteins involved in viral entry, replication, and immune modulation. The F glycoprotein, responsible for fusing the viral and host cell membranes, constitutes the sole antigenic component in recently approved vaccines for older adults and pregnant women (Ruckwardt, 2023; Walsh et al., 2023). Additionally, it is the primary target for prophylactic monoclonal antibodies in preterm and high-risk infants (Shen et al., 2025).

The RSV envelope G protein is a type II transmembrane glycoprotein comprised of a short cytoplasmic N-terminal region (residues 1–37), a transmembrane domain (residues 38–66), and a heavily glycosylated extracellular domain (residues 67–312) (McLellan et al., 2013). The ectodomain contains two highly variable, intrinsically disordered, and extensively N- and O-glycosylated mucin-like regions, which are implicated in immune evasion (Hallak et al., 2000). These regions are separated by a central conserved cysteine-rich domain (CCD) and a heparin-binding domain (HBD) responsible for host cell attachment in immortalized cell lines (Feldman et al., 1999). The CCD is defined by four cysteine residues forming a cysteine noose stabilized by two disulfide bonds (Cys173-Cys186 and Cys176-Cys182 with a 1–4, 2–3 connectivity) (Gorman et al., 1997). This structure contains the CX3C motif, which interacts with the chemokine receptor CX3CR1 (Johnson et al., 2015; Chirkova et al., 2015). An alternative translation initiation at Met48 yields a secreted G ectodomain (sG, residues 67–298) that retains the CCD, HBD, and mucin-like regions. The sG form is proposed to act as an immune decoy by capturing neutralizing antibodies and modulating CX3C chemokine signaling, thereby contributing to pathogenesis (Bukreyev et al., 2008, 2012). While structural differences between sG and the membrane-bound G (mG) are not fully elucidated, available evidence indicates that mG can be detected as species with different apparent molecular weights, depending on the cellular context in which it is produced. These differences may reflect variations in glycosylation patterns and/or the formation of higher-order assemblies through intercatenary covalent interactions (King et al., 2021; Kwilas et al., 2009). Disulfide linked complexes between F and G proteins have been reported (Low. F y G oligomerize in membrane, 2008; Arumugham et al., 1989). These findings position the conserved cysteine motif in the RSV G protein as a candidate redox-active motif, which can dynamically undergo disulfide exchange reactions to yield disulfide-linked hetero-oligomerization between the membrane-bound G and F proteins. This redox-dependent assembly could critically influence viral membrane complex stability, receptor engagement, or immune evasion.

Substantial evidence identifies the CCD as a pivotal virulence factor, facilitating viral entry (Johnson et al., 2015), modulating host immune responses, and contributing to disease pathogenesis (Harcourt et al., 2006). The CCD mediates initial cell surface attachment via the CX3C motif’s interaction with CX3CR1 on human airway epithelial cells (Johnson et al., 2015). This interaction triggers the production of RANTES, IL-8, and fractalkine, while suppressing IL-15, IL1-RA, and monocyte chemotactic protein-1, thereby impairing the local immune response (Chirkova et al., 2015). The CCD’s CX3C motif reduces antiviral T cell responses, affecting CX3CR1+ T cell trafficking to the lung and diminishing populations of crucial immune cells such as IFN-*γ*-producing CD8+ T cells and IL-4-producing cells (Harcourt et al., 2006). Furthermore, CCD: CX3CR1 binding on dendritic cells impairs their maturation and lymph node migration, suppressing type I/III interferon and other pro-inflammatory cytokines (Chirkova et al., 2013). By inhibiting NF-κB nuclear translocation, RSV G reduces cytokine production in monocyte-macrophages, which correlates with decreased lung inflammation in mice during early infection (Polack et al., 2005). This inhibitory effect, characteristic of both RSV subgroups A and B, is elicited by the RSV G CCD and depends on its conformation rather than on exact sequence conservation between subgroups (Polack et al., 2005). Recent evidence has shown that although recombinant sG fails to activate CX3CR1 signaling in monocytic cells, it acts as a competitive antagonist by blocking CX3CL1-mediated activation, and this interaction reduces monocyte migration and adhesion (Meineke et al., 2024). Disrupting the CX3C: CX3CR1 interaction—either by mutating the CCD or using anti-CCD antibodies—restores immune responses (Boyoglu-Barnum et al., 2017; Miao et al., 2009; Haynes et al., 2009; Shingai et al., 2008). In mouse models of RSV infection, recombinant viruses lacking a functional CX3C motif induce a Th1-biased immune response and higher antibody titers compared to wild-type viruses, with reduced pathology (Boyoglu-Barnum et al., 2017; Ha et al., 2019). In addition to CX3CR1-dependent mechanisms, the fully glycosylated G protein has been reported to exert immunosuppressive effects through interactions with the C-type lectin receptor DC-SIGN and L-SIGN on dendritic cells, further contributing to the modulation of antiviral immune responses (Johnson et al., 2012). Moreover, highlighting the context-dependent nature of G protein–mediated immunomodulation, the extracellular domain of the RSV G glycosylated protein also binds to and activates the TLR2/TLR6 axis, initiating an innate inflammatory response through the induction of several proinflammatory cytokines, including TNF-α (Meineke et al., 2026; Alshaghdali et al., 2021; Segovia et al., 2012).

Notably, the same CCD region is immunogenic and can elicit neutralizing antibody responses. The CX3C motif induces strong antibody production, and anti-G monoclonal antibody therapies have been shown to reduce inflammation and disease severity in RSV-infected models by suppressing Th2-polarized cytokines and chemokines while enhancing interferon responses (Lee et al., 2024; Bergeron et al., 2023a,b; Boyoglu-Barnum et al., 2013). Thus, the CCD is both immunomodulatory and immunogenic, raising the critical question of whether these seemingly opposing functions overlap at the sequence or structural level. The G protein and particularly its CCD containing the CX3C motif have been extensively investigated as vaccine targets. However, vaccines based on recombinant glycosylated sG have failed to induce robust neutralizing antibody responses and have led to enhanced lung pathology upon RSV challenge, including eosinophilic and neutrophilic infiltration and elevated Th2 cytokines/chemokines (Kawahara et al., 2024). In contrast, unglycosylated sG produced in *E. coli* induced higher titers of neutralizing antibodies and conferred protection without enhanced pathology (Fuentes et al., 2015). Various nanoparticle, microparticle, and vector-based vaccine platforms displaying the CCD have demonstrated protective efficacy in mice (Jorquera et al., 2013; Voorzaat et al., 2024), yet some CX3C-targeted vaccines have been linked to vaccine-enhanced disease (VED) in animal models. Strategies to mitigate VED include adjuvantation with TLR2 or TLR4 agonists (Bergeron et al., 2023c; Powell et al., 2022) or co-delivery with IL-35 (Yang et al., 2020). The challenge remains to elicit protective immunity without inducing VED, a complication seen in early vaccine trials with formalin-inactivated RSV (Kim et al., 1969). Recently approved RSV vaccines for older adults and pregnant individuals, primarily targeting the prefusion F protein, have demonstrated that robust neutralizing antibody responses confer protection without overt immunopathology (Topalidou et al., 2023; Alandijany and Qashqari, 2025). In these populations, pre-existing immunity enables rapid and balanced protective responses. In contrast, newborns are immunologically naïve and require precise immune priming to establish effective protection while avoiding VED. Understanding the molecular determinants of immunomodulation and inflammation, particularly the RSV G CX3C motif, is essential for developing safe and effective RSV vaccines (Ruckwardt et al., 2019; Mazur et al., 2018; Boyoglu-Barnum et al., 2019). Moreover, identifying the factors that steer immune responses toward protective Th1 rather than Th2 phenotypes is critical to overcoming this paradox.

While the RSV G CCD has been linked to both immunomodulatory and immunogenic functions, the existence of membrane-bound, disulfide-linked F-G complexes suggests its conserved cysteine motif is not merely structural but functionally redox-active. In this study, a peptide (Gpep) comprising the RSV CCD was recombinantly expressed and purified. Its oxidative folding, putative native conformation, and other possible conformations were characterized by uncovering a redox-based structural plasticity. Functional effects of native Gpep were assessed *in vitro* using mouse bone marrow-derived dendritic cells and splenocyte-derived cells. The results indicated that Gpep suppresses TLR4 agonist-induced dendritic cell activation, inhibits MHC class II-presented OVA peptide-induced lymphocyte activation, and reduces human neutrophil activation under various stimuli. Disulfide-driven oligomerization of Gpep abolished its immunosuppressive effects, demonstrating that this function is dependent on the redox conformation of the CCD. This functional redox-switch may reflect a key difference between the soluble and membrane-bound forms of the G protein. We also show that during early RSV exposures in infancy, antibody responses are biased toward the F protein, delaying robust anti-G immunity—a delay we propose is actively shaped by the immunosuppressive properties of Gpep. The study aims to elucidate the immunomodulatory and immunogenic roles of the redox-active RSV G protein CCD, providing information relevant to RSV immunobiology and vaccine development.

## 2 Materials and methods

RSV G peptide expression and purification: The central non-glycosylated region (Gpep, amino acids 149–196) of the RSV strain A2 G protein (UniProt accession number P03423) was subcloned into a pMalE vector as an Nt-MBP tag fusion protein. A TEV protease recognition site was added between the Nt-MBP and Gpep-Ct sequences. The construct was confirmed by Sanger sequencing and further transformed into *E. coli* C41 (DE3) cells. Transformed bacteria were grown overnight (ON) at 37 °C and 220 rpm in LB medium containing 100 μg/mL ampicillin. An ON culture (OD_600nm_ = 3.0) was diluted 1:100 into fresh LB medium and grown for 3 h until OD_600nm_ reached 0.6. Protein expression was induced by adding 0.25 mM isopropyl *β*-D-1-thiogalactopyranoside (IPTG), and the culture was grown for 16 h at 30 °C. Bacterial cells were pelleted at 3,500 rpm for 15 min at 4 °C. The resulting pellet was resuspended in lysis buffer (25 mM Tris–HCl, pH 7.6, 200 mM NaCl). Resuspended cells were lysed by sonication and centrifuged at 20,000 rpm for 20 min at 4 °C. The soluble protein was precipitated by adding ammonium sulfate to a final concentration of 80% with continuous stirring at 4 °C overnight. The precipitate was collected by centrifugation at 12,000 rpm for 20 min at 4 °C and resuspended in 50 mL/L of culture in amylose buffer A: 20 mM Tris–HCl, pH 7.6, 200 mM NaCl. The resuspended sample was loaded onto an amylose affinity purification column (Cytiva) equilibrated in buffer A. The column was washed with 10 CV of buffer A, and protein elution was performed by adding 20 mM maltose in buffer A. The purified fusion protein was incubated with TEV protease at a 1:100 mass ratio for 16 h at 20 °C to cleave Gpep from MBP. Considering the distinct isoelectric points of the two proteins (9.5 for Gpep and 5.1 for MBP), the cleaved Gpep was purified using cation exchange chromatography on a Capto S column (Cytiva). The column was equilibrated with buffer A (50 mM sodium phosphate, pH 7.6). The protein sample was previously dialyzed in buffer A and applied to the column. After washing with 5 CV of buffer A, elution was performed by a 10 CV gradient from 0 to 500 mM NaCl. The Gpep was further purified by size exclusion chromatography in a Superdex 75 10/300 column (Cytiva), previously equilibrated in phosphate-buffered saline (PBS, Gibco). Fractions containing the protein of interest were collected and purity was analyzed by Tris-Tricine Gel electrophoresis stained by Coomassie Blue and by RP-HPLC in a C4 column (see subsection RP-HPLC below). Protein concentration was determined spectrophotometrically by using an extinction coefficient of 5,500 M^−1^ cm^−1^.

Flagellin expression and purification. The central domain of flagellin (amino acids 54–230, UniProt accession number E0U497) was subcloned into a pET-28A+ vector containing a C-terminal 6 × His-tag and confirmed by Sanger sequencing. The construct was transformed into *E. coli* Rosetta2 cells. Bacterial expression was performed under the same growth and induction conditions described above, except that LB medium was supplemented with 100 μg/mL kanamycin. Briefly, cultures were grown to OD_600nm_ ≈ 0.6 and induced with 0.25 mM IPTG for 16 h at 30 °C. Cells were harvested, lysed in 25 mM Tris–HCl pH 7.6, 200 mM NaCl, and clarified by centrifugation. The soluble fraction was purified by immobilized metal affinity chromatography on a Ni^2+^ column (Cytiva) equilibrated with buffer A (20 mM sodium phosphate pH 7.5, 50 mM NaCl, 20 mM imidazole), and eluted with 500 mM imidazole. The eluted protein was extensively dialyzed against PBS.

RP-HPLC. Protein samples were analyzed by reverse-phase high-performance liquid chromatography (RP-HPLC) using a C4 column. Separation was performed with a linear gradient of 20–40% acetonitrile in 0.1% trifluoroacetic acid (TFA) over 50 min at a flow rate of 1 mL/min. Elution was monitored using an in-line UV detector set at 280 nm, and chromatograms were recorded as absorbance versus time. Peak areas were integrated using the manufacturer’s chromatography software.

Thiol quantification. Free thiols in the native or reduced protein sample were quantified using 5,5′-dithiobis(2-nitrobenzoate) (DTNB Ellman, 1959), which was purchased from Sigma Aldrich and used following the manufacturer’s protocol. The sample was reduced via incubation with 1 mM DTT for 1 h, and then desalted with a PD10 column. Calibration was performed with reduced glutathione (GSH, one thiol/molecule).

Oxidative Folding. The peptide at 1.6 mg/mL was reduced and denatured by incubating for 16 h at 20 °C in 50 mM Tris–HCl pH 8.0 containing 100 mM DTT and 6.0 M guanidinium chloride. The sample was buffer exchanged in a PD-10 column in 50 mM Tris–HCl pH 8.0. The folding reaction was allowed to proceed under four different conditions: A (Control-): 50 mM Tris–HCl pH 8.0; B (Control+) 50 mM Tris–HCl pH 8.0 containing 0.25 mM *β*-mercaptoethanol as a thiol initiator, C (GSSG): 50 mM Tris–HCl pH 8.0 containing 1.0 mM GSSG or D: redox buffer (GSSG: GSH mixture at a 1:0.5 mM ratio). The folding reaction was stopped by acidification with 1% TFA at different time points (0, 30, 60, 120, and 240 min for control and β-mercaptoethanol conditions; 0, 1, 2, 4, 8, 16, and 32 min for glutathione-containing conditions). Folding intermediates were analyzed by RP-HPLC, and time-dependent relative abundances were fitted to a sequential two-step irreversible kinetic model:


Red→k1Int→k2Nat

The corresponding system of differential equations is:


d[Red]dt=−k1[Red]d[Int]dt=k1[Red]−k2[Int]d[Nat]dt=k2[Int]

Analytical solutions used for fitting were:


[Red](t)=e−k1t[Int](t)=k1k2−k1(e−k1t−e−k2t)[Nat](t)=1−[Red](t)−[Int](t)

Experimental data were globally fitted to these equations using non-linear least squares optimization implemented in Python (SciPy). Rate constants (k_1_ and k_2_) are reported as best-fit values ± standard errors obtained from the covariance matrix.

Fluorescence spectroscopy. Tryptophan emission fluorescence of Gpep was measured on a fluorescence spectrophotometer (Jasco FP-8500) at 20 °C. The spectra were taken in the 310 to 450 nm interval using a 280 nm excitation wavelength (5 nm excitation and emission slits, 0.1 s averaging time). To evaluate disulfide reduction and peptide denaturation probed by its single Trp residue, samples of Gpep at 20 μM were incubated for 16 h at 20 °C in 20 mM Tris–HCl pH 8.0 containing the following additives: a) no additive; b) 10 mM DTT; c) 6.0 M guanidinium chloride or d) 10 mM DTT and 6.0 M guanidinium chloride. The experiments were performed in duplicates twice.

Western Blot. Protein samples were separated by SDS–PAGE under reducing conditions and transferred onto PVDF membranes (Millipore). Membranes were blocked for 1 h at room temperature (RT) with 1% BSA in TBS-0.1% Tween 20 (TBS-T). After washing, membranes were incubated overnight at 4 °C with a rabbit polyclonal anti-G antibody (SinoBiological, catalog number 11070-RP02) diluted in blocking buffer. Following three washes with TBS-T, membranes were incubated for 1 h at RT with IRDye® 680RD goat anti-rabbit IgG (LI-COR, catalog number 926–68071) diluted in blocking buffer. Fluorescent signals were detected using the Odyssey Imaging System (LI-COR) according to the manufacturer’s instructions.

Molecular Dynamics Protocol. Both oxidized and reduced initial atomic coordinates of Gpep were generated from the PDBid 6BLI (Jones et al., 2018). After removing crystallographic waters and antibody fragments, hydrogen atoms were added by using the *tleap* module of the AMBER18 suite (Case et al., 2018), assigning protonation states consistent with physiological pH. The initial coordinates were solvated in a truncated-octahedral TIP3P water box extending 12 Å beyond the protein. Counterions were added to neutralize the total charge. Energy minimization and molecular dynamics were performed using the ff14SB force field (Maier et al., 2015). Briefly, a standard MD protocol was employed (Patodia, 2014): a two-step minimization, followed by a 0.5 ns in the canonical ensemble (constant number of particles, volume and temperature) equilibration in which the system was heated to 300 K using the Berendsen thermostat, and subsequently switched to the isothermal-isobaric (constant number of particles, pressure and temperature) conditions to equilibrate the density to ~1 g cm^−3^ using the Monte Carlo barostat. A 10 Å cutoff was used for non-bonded interactions, and long-range electrostatics were treated under periodic boundary conditions (PBC) with the particle-mesh Ewald (PME) procedure using a grid spacing of 1 Å (Essmann et al., 1995). The SHAKE algorithm was applied to constrain bonds to hydrogen atoms, and a 2-fs integration time step was used (Ryckaert et al., 1977). After 100 ns of conventional MD, 5 replicas of 200 ns long accelerated molecular dynamics (aMD) simulations were performed to enhance sampling, following the dual-boost scheme (Hamelberg et al., 2004). In all cases, the potential-energy boost did not exceed 5% of the total potential-energy or dihedral contributions. The measured properties of interest were reweighted by approximating the total ΔV using a 10th-order Maclaurin series (Miao et al., 2014). All simulations were carried out with the AMBER18 package (Case et al., 2018), and visualization and molecular graphics were generated using VMD 1.9.1 (Humphrey et al., 1996).

Detoxification. The purified protein was treated with Pierce High-Capacity Endotoxin Removal Resin (Thermo Scientific) according to the manufacturer’s protocol. Polymyxin B was used as an additional control to rule out endotoxin-mediated effects.

Mice. BALB/cJ, C57BL/6 J, and OT-II [B6. Cg-Tg(TcraTcrb)425Cbn/J] breeding pairs were obtained from The Jackson Laboratory (Bar Harbor, ME) and bred in our facilities. The OT-II genotype was verified annually by flow cytometry through detection of the transgenic MHC class II–restricted TCR specific for the OVA peptide. The Ethics Committee Board of the Universidad Nacional de San Martín approved all procedures involving animals.

Immunizations. 6–8-week-old female BALB/cJ mice (*n* = 5 per group) were subcutaneously vaccinated in the scruff of the neck with 200 μL of PBS containing 2 μg of Gpep or control antigen (flagellin), in the presence or absence of Alum (50% V/V). Immunizations were administered on day 0, followed by submandibular blood collection on day 14. A booster dose was given on day 21, with another blood collection on day 35. The third immunization was performed on day 42, and the final blood sample was taken on day 52.

Bone Marrow Derived Dendritic Cells activation assay. Bone marrow was obtained from femurs and tibias of 6- to 10-week-old C57BL/6 J male mice, which were euthanized by cervical dislocation, by flushing RPMI 1,640 medium through the bone interior. The cell suspension was washed, and red blood cells were lysed with red blood cell lysis buffer (Sigma-Aldrich). Cells were cultured at 37 °C and 5% CO_2_ in six-well plates (1.5 × 10^6^ cells per well) in 2 mL RPMI 1640 containing 10% FBS, 50 μg/mL gentamicin (Sigma-Aldrich), and 20 ng/mL of recombinant GM-CSF (GenScript). Media was renewed on days 3 and 5, then cells were harvested on day 8. BMDCs were treated for 18 h with 500 ng/mL LPS and/or 250 nM, 500 nM, or 1,000 nM Gpep and the expression of DC and activation markers were analyzed by flow cytometry. For surface staining, BMDCs were pretreated with TruStain FcX™ PLUS (BioLegend, San Diego, CA, USA, catalog number 156603) for 10 min before antibody staining for 30 min at 4 °C. Antibodies used were: CD11c-APC, clone N418; (BioLegend, San Diego, CA, USA, catalog number 117301); CD86-PE, clone PO3 (BioLegend, San Diego, CA, USA, catalog number 105105); CD80-PEcy7, clone 16-10A (BioLegend, San Diego, CA, USA, catalog number 104734); MCH II-FITC, clone M5-114.1 (BioLegend, San Diego, CA, USA, catalog number 107606). Data acquisition was performed using a BD LSRFortessa™ X-20 cytometer (BD Biosciences, Franklin Lakes, NJ, USA), and data were analyzed with FlowJo software (v10.8.1; FlowJo LLC, Ashland, OR, USA). Debris was excluded based on forward scatter (FSC) and side scatter (SSC) parameters, and doublets were excluded using standard singlet discrimination gating. BMDCs were defined as CD11c^+^ cells, and the expression levels of CD80, CD86, and MHC II were analyzed.

Costimulation assays. For costimulatory assays, splenocytes (3 × 10^5^ cells) from OTII mice were cultured for 48 h at 37 °C in 5% CO_2_ in flat -bottom, 96 -well plates in 0.2 mL RPMI 1640 medium (Thermo Fisher Scientific, Waltham, MA, USA), in the presence of 10% FBS (Gibco, Thermo Fisher Scientific, Massachusetts, USA), 2 mM glutamine, and 5 mg/mL Gentamicin (complete medium), in the presence of the cognate OVA_323–339_ peptide (Sigma-Genosys, The Woodlands, TX, USA) and LPS (Sigma) at the indicated concentrations (500 ng/mL LPS and 0.1 μg/mL OVA_323-339_). To assess proliferation, 1 μCi [3H] methylthymidine (New England Nuclear, Newton, MA, USA) was added to each well 18 h before harvesting. For IFN-*γ* quantification, supernatants were collected 24 h after stimulation, and IFN-γ was measured using Mouse IFN-γ ELISA Kit, (BioLegend, catalog number 430803). All assays were performed in quadruplicate.

Adult blood samples and ethical statement: Blood samples were obtained from healthy volunteer donors who had not taken any medication for at least 10 days before the day of sampling. Blood was obtained by venipuncture of the forearm vein and was drawn directly into plastic tubes containing 3.8% sodium citrate (Merck). This study was performed according to institutional guidelines (National Academy of Medicine, Buenos Aires, Argentina) and received the approval of the institutional ethics committee (N° 10/24/CEIANM), and written informed consent was provided by all the subjects.

Viral stocks and inactivation: Semiconfluent monolayers of HEp-2 cells were infected with RSV strains line A2 (multiplicity of infection = 0.2) and were incubated 3–4 days, monitoring the development of cytopathic effect (CPE) daily, until CPE ≥ 80% of cell monolayer, but still intact and attached to flask bottom. Then, supernatant was removed and 5 mL of cold 25% (w/v) sterile sucrose was added. Then the flask was transferred to −80 °C, being sure that the cell surface is covered with sucrose solution while in the freezer. After three cycles of freezing and thawing, lysates were transferred to sterile 50 mL conical tubes. Cellular debris was removed by centrifugation at 500×*g* and 4 °C for 10 min, and supernatants were aliquoted and stored at −80 °C until use. UV-inactivated virus was prepared from the same virus stock by irradiation in a UV cross-linker for 15 min.

Polymorphonuclear neutrophil isolation: Polymorphonuclear neutrophils (PMN) were isolated by Ficoll–Hypaque gradient centrifugation (Ficoll Pharmacia, Uppsala; Hypaque, Wintthrop Products, Buenos Aires, Argentina) and dextran sedimentation, as previously described (Böyum, 1968). Contaminating erythrocytes were removed by hypotonic lysis. After washing, the cells (96% neutrophils on May Grünwald/Giemsa-stained Cyto-preps) were suspended in RPMI 1640 supplemented with 2% heat-inactivated fetal calf serum (FCS) and used immediately after.

Chemotaxis assay: Chemotaxis was quantified using a modification of the Boyden chamber technique (Betsuyaku et al., 1999). A cell suspension (50 μL) containing 2 × 10^6^ cells/ml in RPMI with 2% FCS, was placed in the top wells of a 48-well micro-chemotaxis chamber. A PVP-free polycarbonate membrane (3 μm pore size; Neuro Probe Inc. Gaithersburg MD, USA) separated the cells from lower wells containing either RPMI or the chemotactic stimulus (Gpep 1 μM or fMLP 10^−7^ M). The chamber was incubated for 30 min at 37 °C in a 5% CO_2_ humidified atmosphere. After incubation, non-migrated cells were gently removed from the upper surface of the membrane. The membrane was then fixed and stained with TINCION-15 (Biopur SRL, Rosario, Argentina). Migrated neutrophils adherent to the lower surface of the filter were counted in five randomly selected high-power fields (HPF) (×400) per well. Results are expressed as the mean number of PMN per HPF from triplicate determinations.

CD11b expression: Neutrophils (5 × 10^5^) were incubated in the presence of Gpep 1 μM, LPS (0.5 ng/mL) or both for 30 min at 37 °C in 5% CO_2_. Immediately after, cells were incubated with a specific mouse anti-human CD11b antibody conjugated with phycoerythrin (PE) (Dako, Santa Clara, CA, USA catalog number 301306). After washing, samples were analyzed by flow cytometry using a FACSCanto cytometer (BD Biosciences, San Jose, CA, USA). Debris was excluded based on forward and side-scatter parameters and CD11b expression was analyzed within the gated-viable neutrophils. Mean Fluorescence Intensity (MFI) of the CD11b was determined on 50.000 events. Data was analyzed using FlowJo v10.8.1 software.

ROS production: ROS production was measured by conventional (non-spectral) flow cytometry using a FACSCalibur instrument (BD Biosciences). Dihydrorhodamine-123 (DHR, Sigma-Aldrich) fluorescence was detected in the FL1 channel, using excitation at 488 nm and emission collected at 530/30 nm. The final data analysis was performed exclusively on FL1 fluorescence intensity, corresponding to oxidized DHR. Briefly, isolated neutrophils (5 × 10^5^) were incubated 15 min at 37 °C with 1 μM DHR. Subsequently, cells were incubated with Gpep 1 μM, fMLP 10^−7^ M or both for 30 min at 37 °C in 5% CO_2_. Immediately after, the green fluorescence was determined by flow cytometry. Data were acquired on a FACSCanto cytometer (BD Biosciences, San Jose, CA, USA) and analyzed using FlowJo v10.8.1 software.

Degranulation assay: To evaluate primary o azurophilic granule mobilization, neutrophils (1 × 10^6^) were incubated in a 24-well plate with Gpep 1 μM, PMA 20 nM (Merck) or both for 2 h at 37 °C in 5% CO2. The MPO release was evaluated in cell-free supernatants by measuring its activity. MPO activity was determined using the specific substrate 3, 3′, 5, 5′-tetramethyl-benzidine (TMB) (Invitrogen, Thermo Fisher) and measuring the resultant absorbance at 450 nm, subtracting the absorbance at 570 nm (450–570 nm).

NETs formation: Neutrophils (1 × 10^6^) were incubated in a 24-well plate in the presence of Gpep 1 μM, PMA 20 nM or both for 3 h at 37 °C in 5% CO_2_. After the incubation period, micrococcal Nuclease S7 (4 U, Roche Diagnostics) was added for 15 min at 37 °C in order to cut and release the DNA associated with NETs from the cell body of neutrophils. Then, inactivation of the enzyme was performed by the addition of 5 mM of EDTA. Supernatants were collected and centrifuged twice, first at 900×*g* and then at 9,600×*g*. To determine the presence of double stranded (ds) DNA, a commercial kit was used (Invitrogen, Thermo Fisher), containing picogreen as the DNA intercalator, which was read in a fluorimeter (DeNovix DS-11 spectrophotometer).

Cell viability: Neutrophils (5 × 10^5^) were incubated with Gpep or a positive control of death (PMA, 100 nM) for 1 or 4 h at 37 °C in 5% CO_2_. After the incubation period, cells were washed and incubated with 7-Amino-Actimycin D (7-AAD; BD Biosciences, 10 μg/mL) for 10 min on ice in the darkness. Immediately after, cell viability was determined by flow cytometry, since 7-AAD is excluded by viable cells but can penetrate cell membranes of dying or dead cells.

Pediatric blood samples. Peripheral blood samples were obtained from pediatric outpatients attending Hospital de Niños “Sor María Ludovica” (La Plata, Buenos Aires, Argentina) during the years 2023 and 2024. Participants were clinically healthy children between 0 and 72 months of age, with no evidence of acute infection at the time of sampling or during the past month. Blood was collected by venipuncture during routine clinical procedures, and serum was separated and stored at −80 °C until use. The study was approved by the Institutional Review Board of the Hospital de Niños Sor María Ludovica de La Plata (Comité Institucional de Revisión de Protocolos de Investigación, CIRPI). Informed consent for serum sample collection was obtained from all participating parents or legal guardians.

Determination of IgG Antibodies Against RSV G or F Proteins by ELISA. Indirect ELISAs were performed using flat-bottom high-binding ELISA plates (Costar, Corning, NY, USA) coated overnight at 4 °C with 50 ng per well of recombinant RSV G or RSV F proteins (SinoBiological, catalog numbers 11,070-V08H and 11,049-V08B). Both proteins were produced in eukaryotic expression systems and correspond to sequences from the RSV A strain. To ensure assay specificity, it was initially validated by comparing serum reactivity against the RSV G protein and an unrelated control protein (MBP), confirming specific and selective recognition of RSV G. Plates were washed three times with washing buffer (TBS + 0.1% Tween-20) and blocked for 1 h at room temperature (RT) with 1% BSA in TBS (Gibco). After discarding the blocking solution, serial dilutions of sera (human or mouse) were added and incubated for 2 h at RT. Plates were then washed three times and incubated with HRP-conjugated secondary antibody appropriate for the species tested: mouse anti-human IgG for human sera, goat anti-mouse IgG for mouse sera (ThermoFisher, Waltham, MA, USA) for 1 h at RT. Following three additional washes, plates were developed with 1-Step Ultra TMB Substrate (ThermoFisher) and the reaction was stopped with 1 N sulfuric acid. Absorbance was measured at 450 nm using a microplate reader (BioTek, Winooski, VT, USA). Antibody titers were calculated by fitting the absorbance values to a four-parameter logistic (4PL) sigmoidal curve using nonlinear regression. The titer was defined as the reciprocal of the serum dilution corresponding to a cutoff value equal to 1.5 times the absorbance of the blank for each plate.

Statistical analysis. Statistical analyses were performed using custom scripts written in Python (3.10) employing the NumPy, SciPy and Pandas libraries. Oxidative folding kinetics were analyzed by fitting the experimental data to a two-step sequential kinetic model (see subsection Oxidative Folding). Comparisons of inflammatory response in BMDCs, splenocytes, and PMN were performed using one-way ANOVA followed by appropriate post-hoc tests when applicable. ELISA titration curves were fitted using a four-parameter logistic (4PL) model, and antibody titers were calculated accordingly using a cutoff value defined as 1.5× the mean blank signal. Paired comparisons of anti-G and anti-F titers in ELISA were analyzed using the Wilcoxon matched-pairs signed-rank test. Comparisons of anti-F/anti-G ratios across multiple age groups were assessed using the Kruskal–Wallis test followed by Dunn’s multiple comparison test. ELISA data from mouse immunization experiments were analyzed by one-way ANOVA with all groups compared to the control condition. Statistical significance was defined as *p* < 0.05.

## 3 Results

### 3.1 The RSV G central cysteine-rich peptide exhibits redox-dependent conformational plasticity and oligomerization

**Figure 1:**
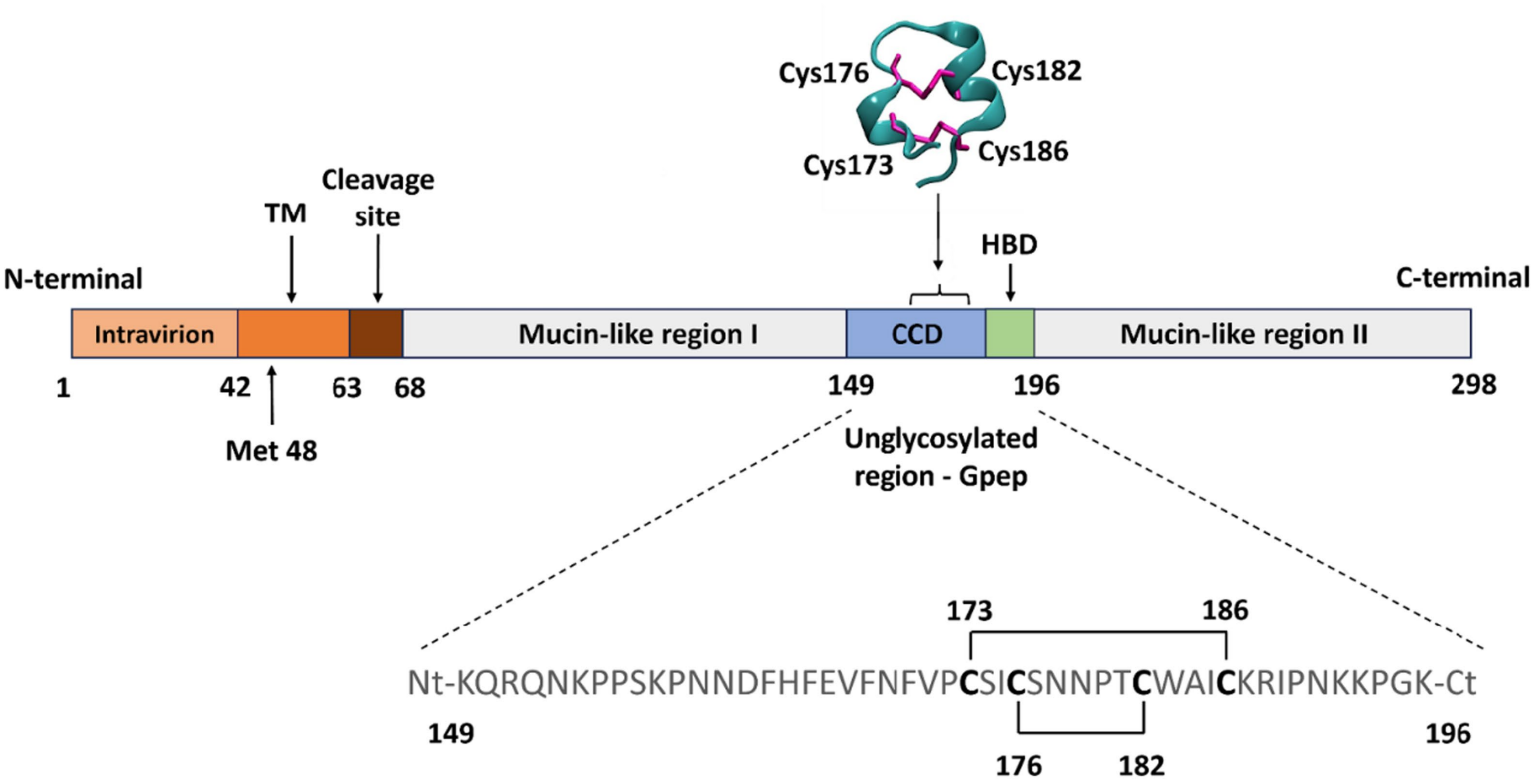
Structure of the full-length RSV G protein and its central conserved region. Schematic representation of the full-length, membrane-bound (mG) RSV G protein, showing its major domains: the N terminal intravirion or cytoplasmic tail, transmembrane domain (TM), cleavage site, and mucin-like regions I and II. Methionine 48 (Met 48), the translation start site for the secreted form (sG), is indicated. The sG protein is generated by proteolytic cleavage, which removes a truncated TM domain, releasing sG from the cell. The central conserved domain (CCD) and heparin-binding domain (HBD) together constitute the unglycosylated region corresponding to the recombinant peptide Gpep analyzed in this work. The sequence and disulfide bond connectivity of Gpep (based on the canonical RSV A2 strain, UniProt accession number P03423) are detailed in the lower panel. The CX3C motif (CWAIC) is located within this conserved cysteine-rich domain. The structure of the cysteine noose (PDB: 6BLH) is represented in the upper panel.

All the accumulated evidence in RSV G points toward the CCD as the critical sequence concentrating immunomodulatory properties, redox-sensitive structural determinants and neutralizing epitopes (Meineke et al., 2024; Bergeron et al., 2023b; Jones et al., 2018). Thus, the central cysteine-rich region of the RSV G protein (Gpep, residues 149–196) containing the CX3C chemokine motif was recombinantly expressed and purified to high homogeneity (Supplementary Figure 1). While structural evidence shows that this region adopts a cysteine noose stabilized by two intrachain disulfide bonds (Cys1-Cys4, Cys2-Cys3) (Figure 1), experimental evidence demonstrates that multimeric forms of membrane-associated G and F proteins can form via intermolecular disulfide bonds (Hamelberg et al., 2004; Miao et al., 2014), revealing redox states beyond the classic cysteine noose (King et al., 2021).

**Figure 2:**
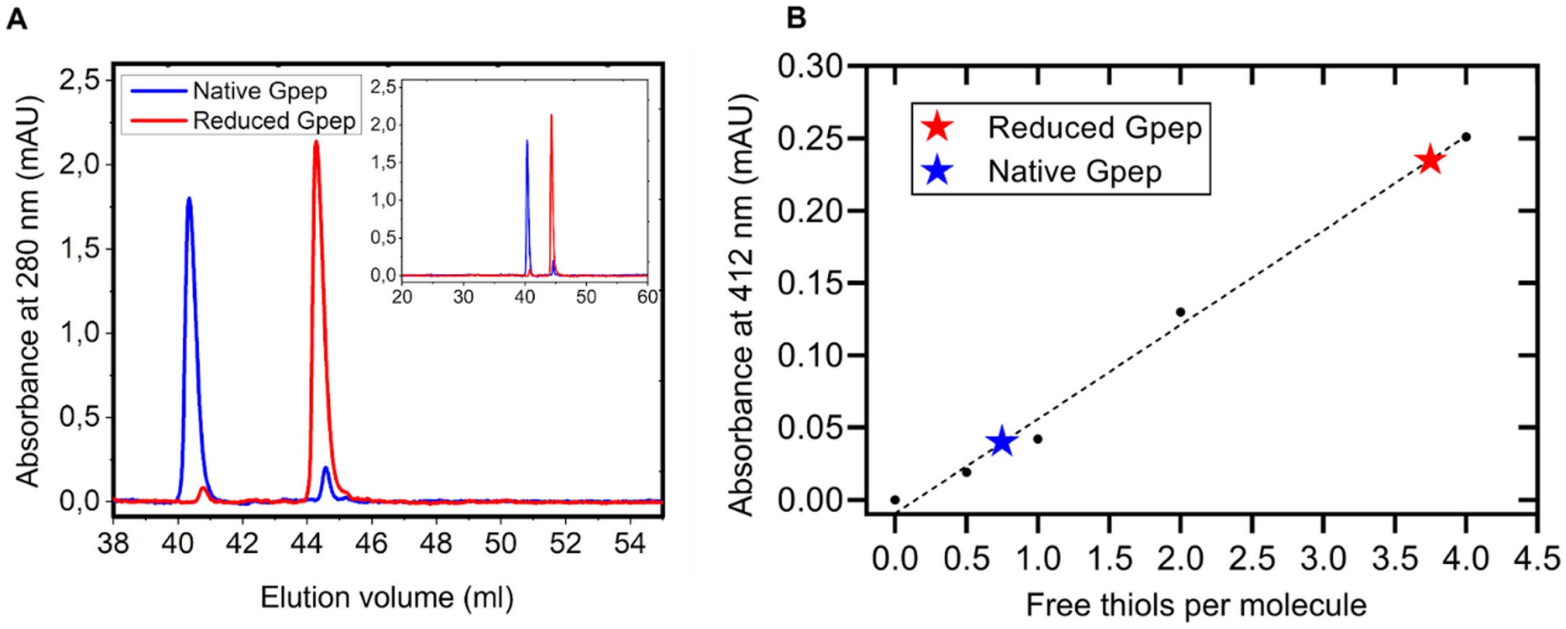
Recombinant Gpep is predominantly oxidized and stabilized by intramolecular disulfide bonds. **(A)** RP-HPLC profile of the native peptide (60 μM, blue trace: elution volume at 40 mL) and its reduced form (60 uM, red trace: elution volume at 44 mL). **(B)** Quantification of free thiols using DTNB (Ellman’s reagent). The native peptide showed ~0.5–1 free thiols per molecule, while the reduced form revealed ~3.5–4 thiols per molecule, supporting the presence of intramolecular disulfide bonds in the native state.

**Figure 3:**
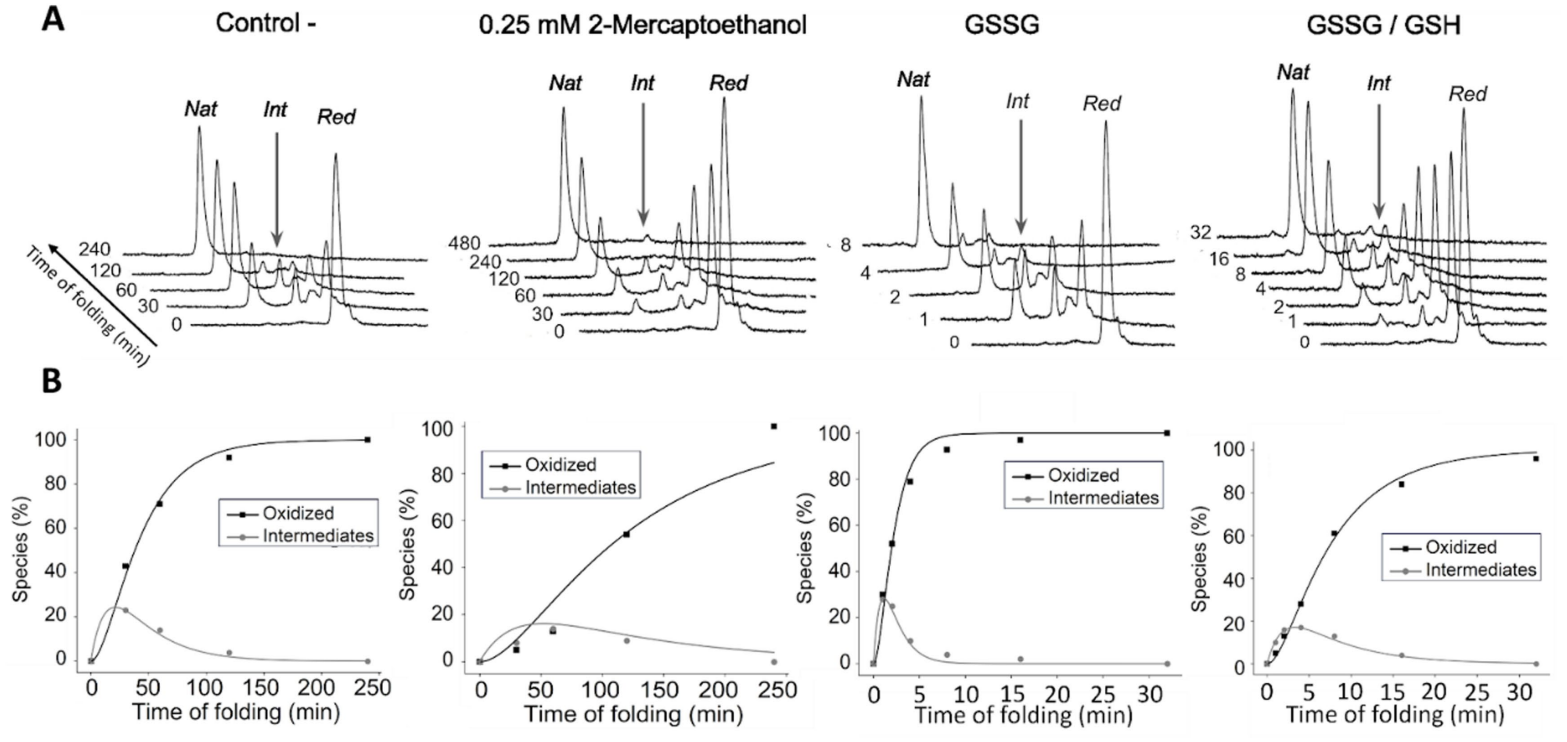
Oxidative folding of Gpep under different redox conditions. Oxidative folding of reduced Gpep was monitored by RP-HPLC in Tris–HCl buffer (pH 8.0) either without additives (Control −) or in the presence of 0.25 mM 2-mercaptoethanol, 0.5 mM oxidized glutathione (GSSG), or a mixture of 0.5 mM GSSG + 1 mM GSH. Each panel is labeled with the corresponding redox condition. **(A)** RP-HPLC chromatographic profiles collected at different time points for each redox condition, showing the evolution of reduced (Red), native oxidized (Nat), and major intermediate (Int) Gpep species. **(B)** Quantitative analysis of the oxidative folding reaction, showing the relative abundance of reduced, intermediate, and oxidized species over time, calculated from peak areas of the chromatograms shown in panel A. The solid lines represent fits to a two-step kinetic model (see Materials and Methods).

To systematically probe the redox landscape of Gpep, we characterized its native and reduced states. RP-HPLC analysis (Figure 2A) revealed a predominant peak corresponding to the native oxidized species, whereas the fully reduced peptide displayed a distinct retention time consistent with increased hydrophobic exposure. Thiol quantification using DTNB (Ellman’s, 1959 reagent for free thiol quantifying) (Figure 2B) showed minimal free thiols in the native preparation (0.5–1 per molecule), compared to the expected four thiols in the fully reduced state, supporting the presence of intrachain disulfide bonds. Refolding assays of reduced Gpep under various redox conditions showed efficient formation of the oxidized species with the accumulation of a major intermediate (I) (Figure 3A). Folding kinetics for each condition were analyzed using a two-step kinetic model (R → I → N), with rate constants k_1_ and k_2_ for the R → I and I → N transitions, respectively. The model was fitted to RP-HPLC peak areas measured under different redox conditions. Representative fits are shown in Figure 3B, and the resulting parameter estimates are listed in Supplementary Table 1. As expected, in the presence of GSSG, K_1_ exhibited a large 21-fold increase, from 0.032 to 0.681 min^−1^ and K_2_ increased 16-fold from 0.066 to 1.108 min^−1^, relative to the Tris pH 8.0 condition. Pairwise comparisons between conditions with and without free thiols (Native vs. *β*-mercaptoethanol and GSSG vs. GSSG + GSH) revealed that the presence of free thiols slowed folding kinetics, with k_1_ reduced by approximately 3 to 4-fold and k_2_ reduced by about 2-fold. These results are consistent with an initial rate-limiting step involving oxidation of the first disulfide bond, which leads to a kinetically trapped intermediate, followed by oxidation of the second disulfide bond. The presence of free thiol agents, known to facilitate disulfide reshuffling, likely led to the formation of non-productive species and a substantial deceleration of the folding kinetics. Despite differences in folding rates and intermediate accumulation, all conditions ultimately yielded an oxidized product indistinguishable from recombinant oxidized Gpep.

**Figure 4:**
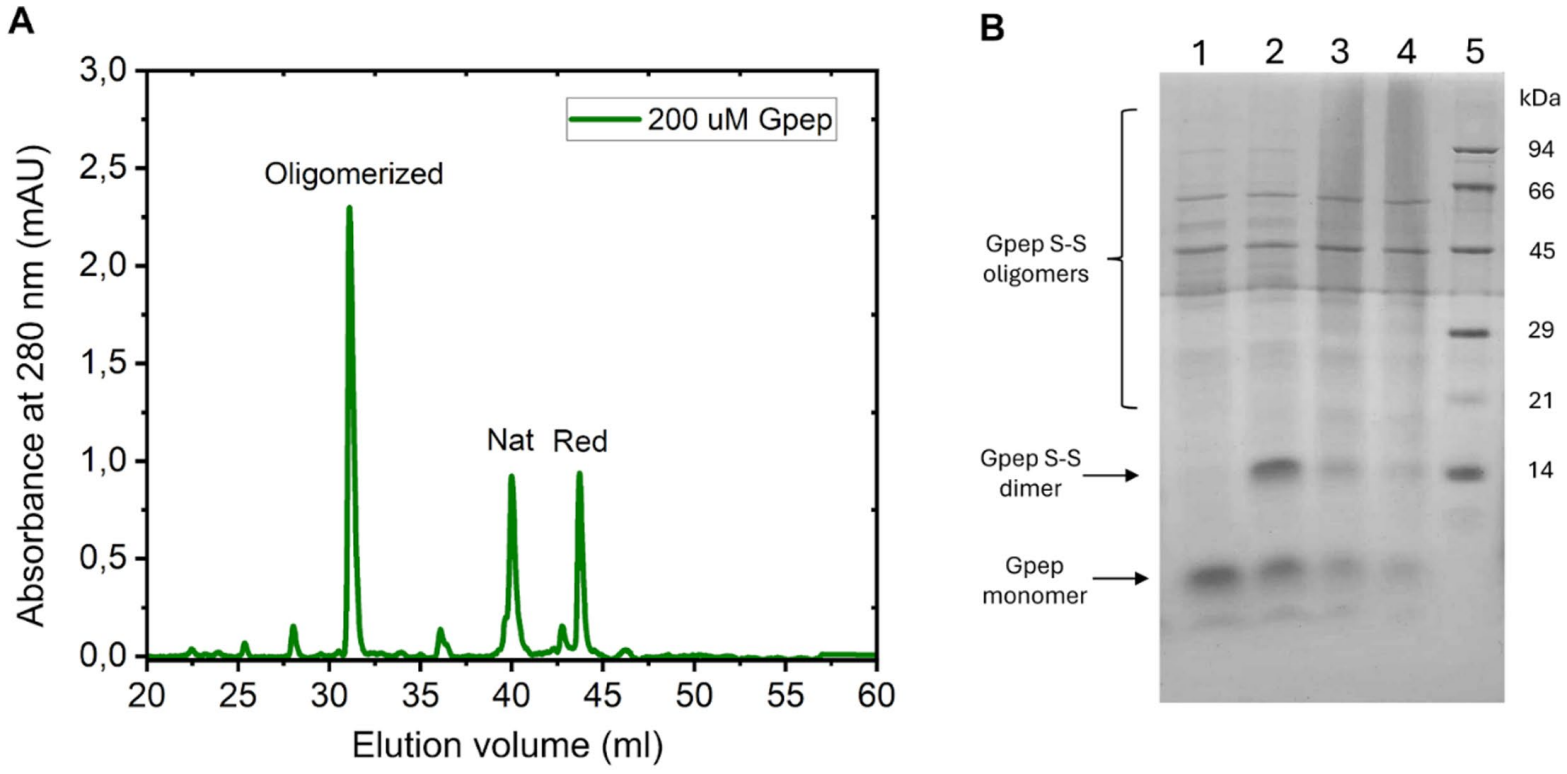
Gpep forms higher-order disulfide-mediated oligomers assembled from highly stable disulfide-linked dimers. **(A)** RP-HPLC profile of Gpep at 200 μM. The major species eluded at 31 mL, corresponding to the oligomerized form. **(B)** Oligomerization state of Gpep analyzed by Tricine SDS–PAGE. Gpep samples were resolved on a 16% Tricine SDS–PAGE gel under the indicated redox and thermal conditions. Lane 1: Gpep treated with *β*-mercaptoethanol and heat-denatured; lane 2: Gpep treated with β-mercaptoethanol without heating; lane 3: heat-denatured Gpep without reducing agent; lane 4: non-reduced, non-heated Gpep; lane 5: molecular weight marker.

Interestingly, when Gpep was concentrated, we observed a shift in the RP-HPLC profiles coinciding with the appearance of higher molecular weight, disulfide-linked oligomers (Figure 4A). These oligomeric species were sensitive to reducing agents, indicating that intermolecular disulfide bonds drive their formation. This observation was further supported by SDS-PAGE analysis performed in reducing and non-reducing conditions, which revealed an ensemble of high-molecular-weight Gpep oligomers that collapse predominantly into a disulfide-linked dimer upon reduction (Figure 4B). Notably, this dimeric species was highly resistant to reduction and disappeared only after heating at 90 °C in the presence of *β*-mercaptoethanol, suggesting that the higher-order oligomers are assembled from exceptionally stable, disulfide-crosslinked dimers. This concentration-dependent oligomerization suggests a structural adaptability of the central region of G, which may offer clues as to how membrane confinement and local concentration might affect the structural differences observed between the soluble (sG) and membrane-bound (mG) forms of the RSV G protein. It must be stressed that this transition is observed only by changing the peptide concentration without any additional redox stimulus. Such plasticity and redox-dependent behavior may have functional implications for RSV biology.

**Figure 5:**
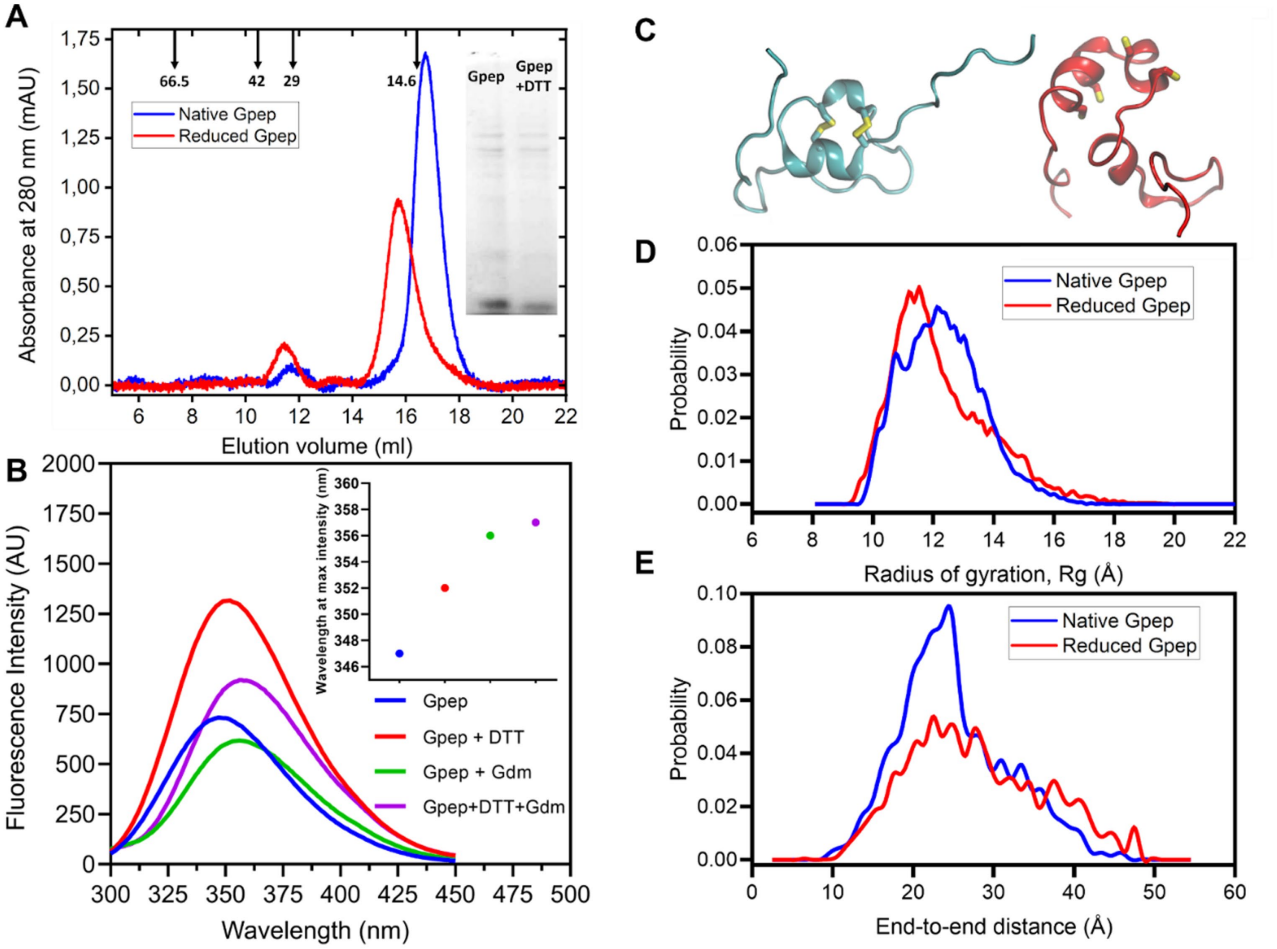
Oxidized Gpep adopts an extended monomeric conformation, whereas the reduced form populates a partially compact state. **(A)** Size-exclusion chromatography of native and reduced Gpep on a S75 10/300 column equilibrated in PBS at 20 °C. According to the column calibration, the apparent molecular mass of native Gpep is ~13.6 kDa and ~16.2 kDa for reduced Gpep. The globular proteins used for column calibration are: BSA (66.5 kDa), MBP (42 kDa), mCherry (29 kDa) and lysozyme (14.6 kDa). Their elution volumes are indicated by an arrow. **(B)** Tryptophan fluorescence emission spectra of 20 μM Gpep treated with: 10 mM DTT (red line), 6.0 M Gdm·Cl (green line), both reagents (violet line) or no additives (blue lines). Inset: Wavelength of maximum emission of each spectrum. **(C)** Representative snapshots of both oxidized (blue) and reduced (red) Gpep models obtained by aMD simulations. The four Cys residues are highlighted. **(D)** Probability distributions of Gpep radius of gyration (*R_g_*, Å) and **(E)** end-to-end distances (Å).

Hydrodynamic (Figure 5A) and structural analyses (SEC, SDS-PAGE, and fluorescence spectroscopy) (Figure 5B) revealed that the oxidized Gpep behaves as an elongated monomer in solution, but reduction leads to a partially compact, flexible conformation. According to the S75 column calibration with globular proteins, SEC analysis revealed a hydrodynamic radius (R_h_) of 1.58 nm for the oxidized protein, which increases to 1.67 nm for the reduced form. This ~6% compaction upon disulfide bond formation is consistent with a reduced Gpep species populating a partially compact, molten globule-like state, while the oxidized form behaves as an elongated monomer in solution. SDS-PAGE analysis (inset of Figure 5A) confirmed that both species migrate predominantly as monomers under denaturing conditions. Intrinsic fluorescence spectroscopy revealed a red shift in the emission maximum upon reduction with DTT or denaturation with guanidinium chloride, indicating increased exposure of hydrophobic residues. Notably, the combined presence of DTT and guanidinium chloride produced a larger shift than either treatment alone, indicating additive effects on conformational expansion and solvent exposure. Molecular dynamics simulations (Figures 5C–E) further supported the subtle difference between the behavior of the reduced and oxidized Gpep, supporting a transition from a molten globule state to an elongated monomer.

### 3.2 The recombinant RSV G-peptide exhibits both immunogenic and immunosuppressive properties

To evaluate the immunogenic potential of the recombinant G central cysteine-rich domain (Gpep), we assessed its ability to present native-like epitopes and elicit functional antibodies. Enzyme-linked immunosorbent assays (ELISA) using sera from healthy adults with prior RSV exposure demonstrated robust recognition of recombinant Gpep with antibody titers comparable to those against full-length, commercial G protein (Supplementary Figure 2A). Paired analysis using a Wilcoxon matched-pairs signed rank test revealed no statistically significant difference between responses to the two antigens. This finding substantiates that Gpep preserves native epitopes reflective of the viral protein. Furthermore, sera from BALB/cJ mice immunized with alum-adjuvanted Gpep elicited a strong anti-G IgG response in an ELISA against commercially glycosylated full-length G protein, with antibody titers exceeding those of control mice by more than three orders of magnitude (Supplementary Figure 2C), validating that the recombinant peptide presents authentic antigenic determinants. Recognition of Gpep by a commercial anti-G antibody in a Western blot confirms the preservation of its native epitopes (Supplementary Figure 2B).

**Figure 6:**
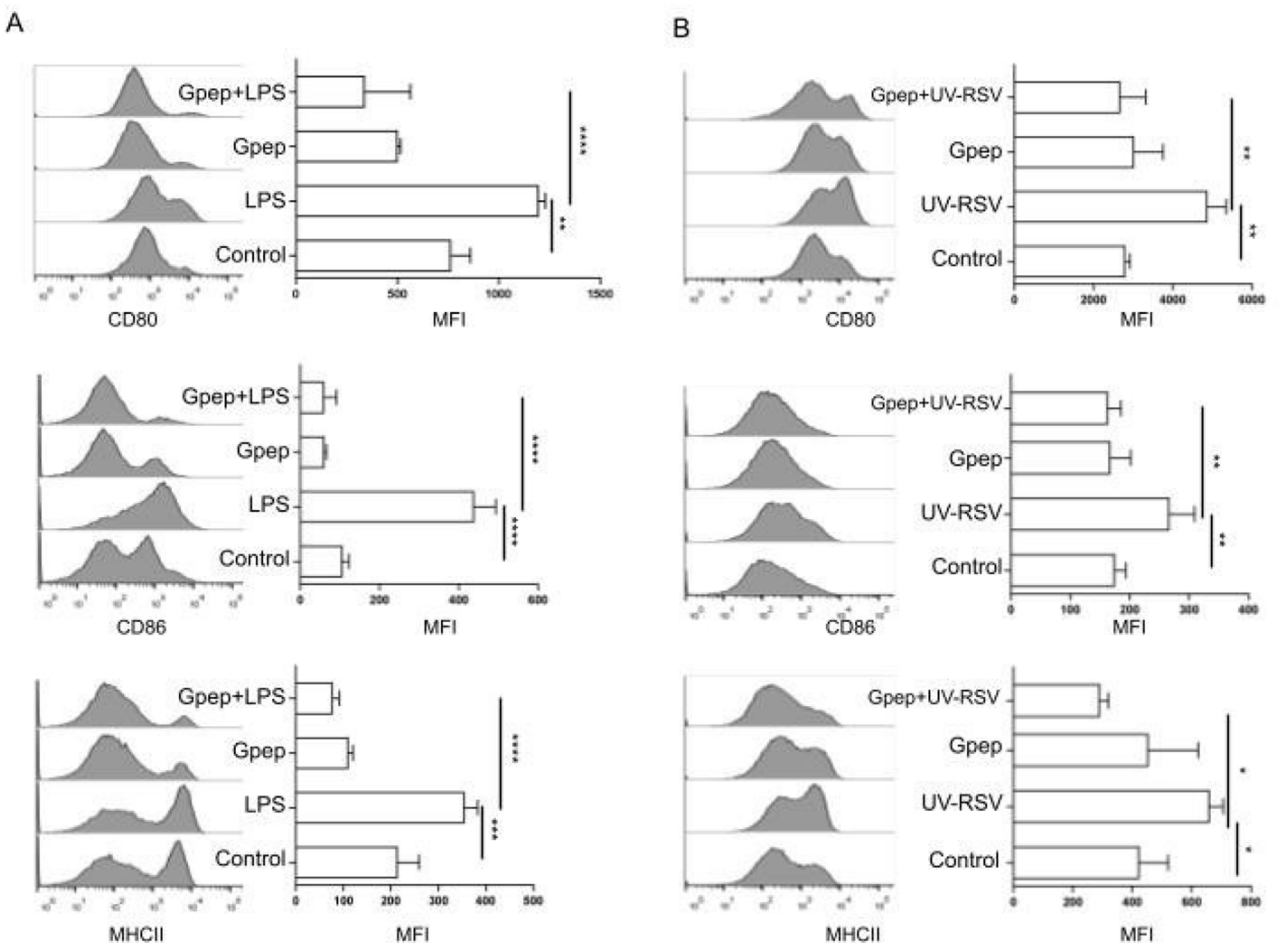
Gpep attenuates LPS and UV-RSV-induced activation of BMDCs. **(A)**. BMDCs were incubated for 18 h at 37 °C in the presence of 500 ng/mL LPS, 1 μM Gpep, or 500 ng/mL LPS plus 1 μM Gpep. Controls are cells without any stimulus. Then, cells were labeled with monoclonal antibodies (mAbs) specific for CD11c, CD80, CD86 and MHCII, and activation was analyzed by flow cytometry in CD11c + cells. **(B)**. BMDCs were incubated for 18 h at 37 °C in the presence of UV-inactivated RSV (UV-RSV) at a MOI of 500, 1 μM Gpep, or UV-RSV at MOI of 500 plus 1 μM Gpep. Control cells were incubated with RSV inactivation media. Then, cells were labeled with monoclonal antibodies (mAbs) specific for CD11c, CD80, CD86 and MHCII, and activation was analyzed by flow cytometry. Representative histograms depict fluorescence intensity corresponding to the expression of CD80, CD86, and MHCII molecules on the CD11c^+^ gated population. Bars represent the geometric mean fluorescence intensity (MFI) ± SD. ^*^p < 0.05, ^**^p < 0.01, ^***^p < 0.001, ^****^p < 0.0001 (ANOVA). Depicted assays are representative of at least two independent experiments.

**Figure 7:**
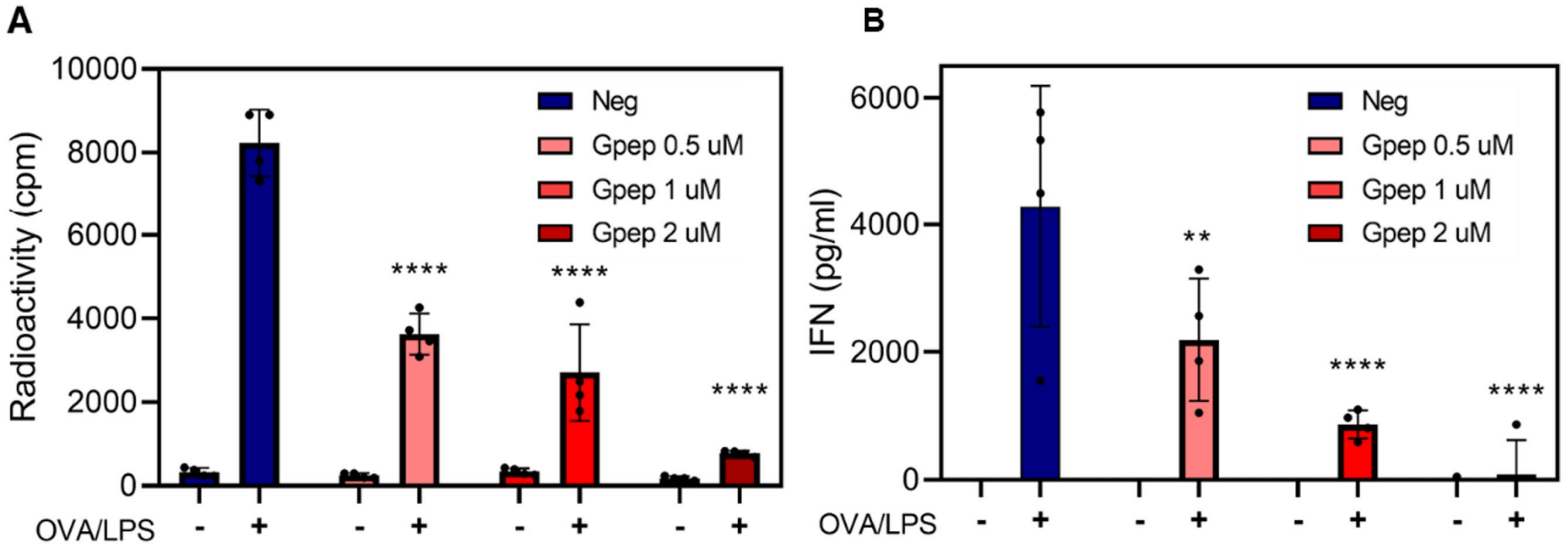
Gpep inhibits splenocyte activation in a dose dependent manner. **(A)** Splenocytes were recovered from the spleens of OT-II mice, red blood cells were lysed, and cells were incubated for 18 h at 37 °C in the absence (−) or presence (+) of activation stimuli (500 ng/mL LPS and 0.1 μg/mL OVA peptide), with Gpep at 0, 0.5, 1, or 2 μM concentrations. At this time point, IFN-*γ* levels in the supernatants were quantified by indirect ELISA. **(B)** Cells were incubated for 48 h in the presence of the stimuli and [^3^H]-methylthymidine. [^3^H]-methylthymidine incorporation was measured to assess cell proliferation. Bars represent the geometric mean ± SD of radioactivity (CPM) or IFN-γ concentration (pg/mL). ^*^p < 0.05, ^**^p < 0.01, ^***^p < 0.001, ^****^p < 0.0001 (ANOVA, all comparisons to Neg). Depicted assays are representative of at least two independent experiments.

Beyond its immunogenicity, the CCD exhibited immunomodulatory activity in mouse bone marrow–derived dendritic cells (BMDCs). Stimulation with LPS or UV-inactivated RSV significantly increased the mean fluorescence intensity (MFI) of CD80, CD86, and MHC-II compared to unstimulated controls (Figures 6A,B). Co-incubation with recombinant Gpep resulted in a significant reduction in MFI of these activation markers relative to the respective stimulated controls, as determined by one-way ANOVA. This indicates that Gpep can modulate innate immune activation under both artificial and physiologically relevant conditions. In ex vivo assays using splenocytes from OT-II transgenic mice, stimulation with LPS and OVA peptide induced robust IFN-*γ* production and proliferative responses, as measured by ELISA (18 h) and [^3^H]-methylthymidine incorporation (48 h), respectively (Figures 7A,B). Addition of Gpep (0.5–2 μM) resulted in a dose-dependent reduction in IFN-γ secretion and cell proliferation. Statistical analysis was performed by one-way ANOVA with comparisons to the unstimulated control, as indicated in the figure. These results demonstrate that Gpep attenuates antigenic specific T cell responses, likely via its effects on dendritic cell function. Importantly, the observed immunomodulatory effects were not attributable to endotoxin contamination, since the absence of biologically active LPS in Gpep preparations was verified by co-incubation with polymyxin B. In contrast to LPS, polymyxin B had no effect on CD80 expression in BMDCs treated with Gpep. Representative flow cytometry histograms comparing Gpep alone and Gpep plus polymyxin B are shown in Supplementary Figure 3.

**Figure 8:**
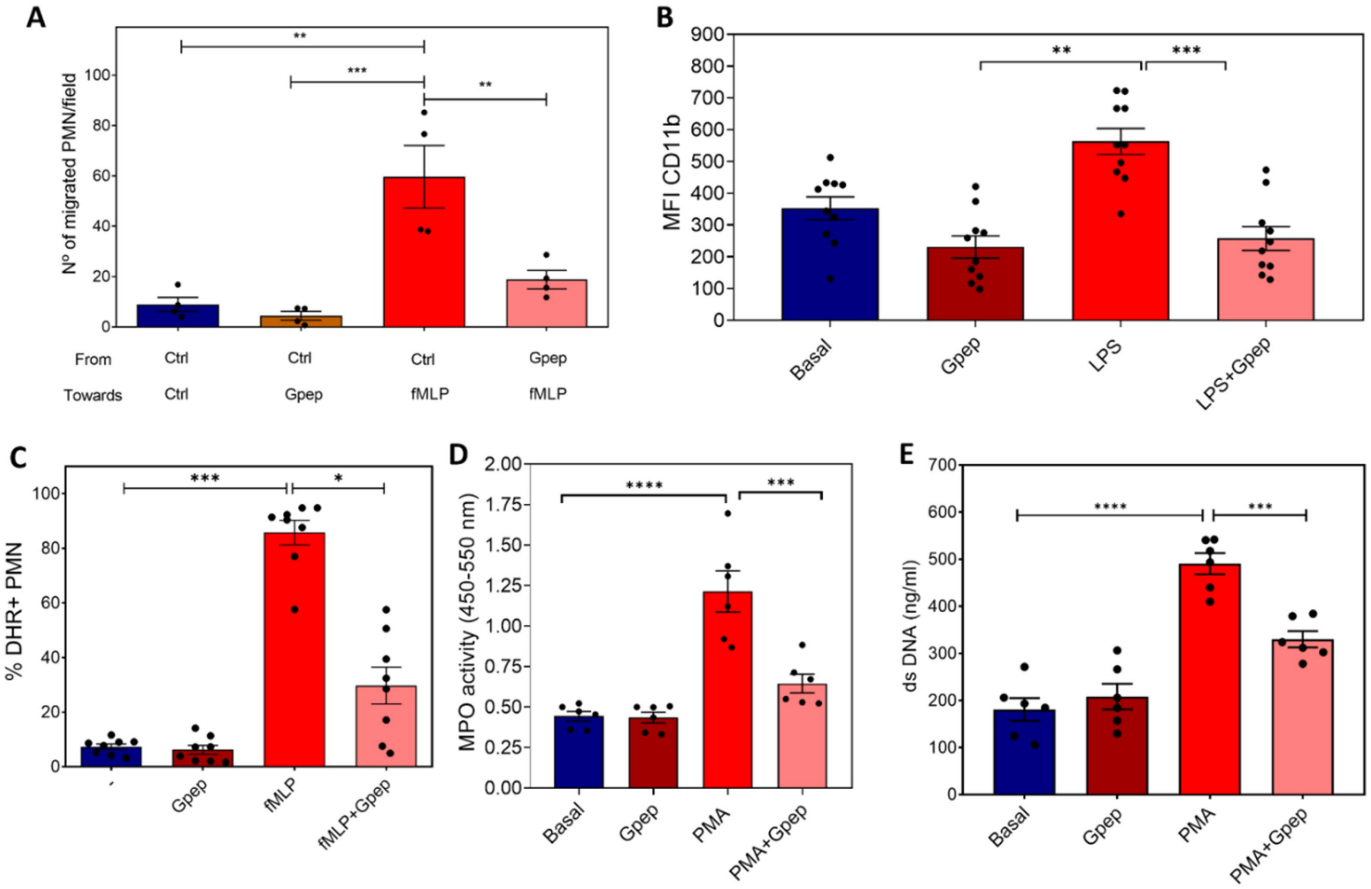
Gpep suppresses multiple functional responses in human neutrophils *in vitro*. **(A)** Neutrophil migration was evaluated using a modified Boyden chamber assay in the presence or absence of Gpep 1 uM. Migrated cells were quantified after 30 min, and results are expressed as PMN per HPF that migrated toward the chemotactic stimulus. **(B)** Surface CD11b expression. Neutrophils were incubated for 30 min with Gpep 1 uM or vehicle and stimulated with fMLP (10^−7^ M). The mean fluorescence intensity (MFI) of CD11b was determined by flow cytometry. **(C)** Reactive oxygen species (ROS) production. Neutrophils were incubated for 30 min with Gpep or vehicle and stimulated with fMLP (10^−7^ M). ROS generation was quantified by flow cytometry using the DHR probe, and results are expressed as the percentage of DHR^+^ cells. **(D)** Myeloperoxidase (MPO) release was quantified in cell-free supernatants collected after 2 h of stimulation, measured spectrophotometrically, indicating primary granule mobilization. **(E)** Neutrophil extracellular trap (NET) formation. Extracellular DNA was quantified after 3 h stimulation, as an indicator of NET release. In all cases, results are expressed as mean ± SEM from at least four donors. ^*^p < 0.05, ^**^p < 0.01, ^***^p < 0.001, ^****^p < 0.0001 by ANOVA, all comparisons to Control + (LPS, fMLP, PMA).

The immunosuppressive activity of Gpep extended to human neutrophils, a cell type implicated in both protective and pathological responses during RSV infection. In a modified Boyden chamber assay, Gpep (1 μM) significantly reduced neutrophil migration toward fMLP after 30 min, quantified as the percentage of input cells (Figure 8A). Gpep also decreased fMLP-induced (10^−7^ M) upregulation of CD11b surface expression, as measured by mean fluorescence intensity (MFI) (Figure 8B; Supplementary Figure 4B), and reduced fMLP-induced reactive oxygen species (ROS) production, quantified as the percentage of DHR^+^ cells (Figure 8C; Supplementary Figure 4A). Furthermore, Gpep attenuated myeloperoxidase (MPO) release after 2 h of stimulation (Figure 8D) and reduced neutrophil extracellular trap (NET) formation after 3 h, assessed by extracellular DNA quantification (Figure 8E). In all assays, statistical comparisons were performed by ANOVA relative to the corresponding stimulated control conditions. The working concentration of Gpep (1 μM) was selected based on preliminary dose–response experiments using neutrophil oxidative burst as a sensitive functional readout, identifying a robust, reproducible response without evidence of saturation or cytotoxicity (Supplementary Figure 4C). Importantly, these inhibitory effects occurred without compromising neutrophil viability (Supplementary Figure 4D), confirming that Gpep specifically attenuates neutrophil activation rather than inducing cytotoxicity. Consistent effects were observed across neutrophils from at least four independent donors, with no exaggerated inter-donor variability or outlier responses.

Notably, the immunomodulatory activity of Gpep was dependent on its oligomerization state. Concentrated Gpep (200 μM vs. 20 μM, Figure 4) that results in an oligomerized form of Gpep, displayed a marked loss of immunomodulatory function within physiologically relevant concentrations (Supplementary Figure 5), paralleling the lack of immunosuppressive activity observed for membrane-bound G (mG) in inactivated RSV. These findings suggest that the oligomeric form of Gpep does not engage in immunomodulation, likely due to conformational constraints that prevent interaction with immune receptors and/or disruption of the CX3C motif. Thus, only the monomeric, native-like form of Gpep is capable of exerting immunosuppressive effects, suggesting a potential functional divergence between soluble and membrane-bound states of the RSV G protein.

### 3.3 Divergent kinetics of anti-G and anti-F IgG antibodies during early Pediatric RSV exposure

To elucidate the humoral immune response dynamics following primary and early exposures to RSV, and to specifically assess the role of the immunomodulatory CX3C motif within the G protein, we quantified IgG antibody titers against the two principal viral antigens, F and G, in sera from a cohort of healthy, ambulatory pediatric patients with no evidence of acute infection at the time of sampling or during the past month. Stratification by age allowed us to distinguish between groups predominantly protected by maternally derived antibodies (0–6 months) and those where endogenous, adaptive immunity increasingly contributes (6–12, 12–24 and 24–72 months).

**Figure 9:**
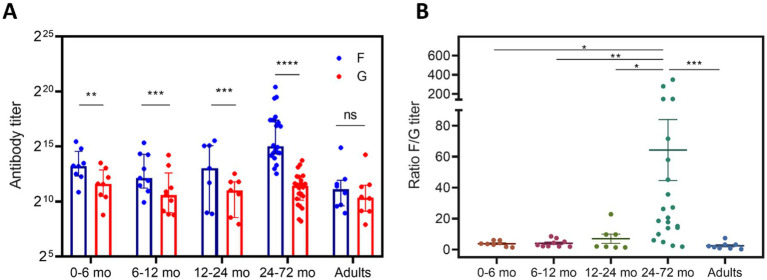
Uncoupling of anti-G and anti-F responses during early exposures to human RSV. **(A)** Anti-F and anti-G antibody titers across different age groups are shown as bar graphs representing median ± interquartile range. Each age group includes paired measurements of anti-F and anti-G titers from individual donors. Paired data were compared using the Wilcoxon signed-rank test. ^*^p < 0.05, ^**^p < 0.01, ^***^p < 0.001, ^****^p < 0.0001, ns, not significant. Sample size per age group: 0–6 mo (8), 6–12 mo (9), 12–24 mo (7), 24–72 mo (22), Adults (8). **(B)** Ratio of anti-F to anti-G titers (F/G ratio) represented as a dot plot, where each dot corresponds to an individual donor. Data were analyzed by Kruskal–Wallis test followed by Dunn’s post-hoc test. The horizontal lines indicate the median value and interquartile range. ^*^p < 0.05, ^**^p < 0.01, ^***^p < 0.001, ^****^p < 0.0001, ns, not significant.

Analysis of antigen-specific antibody titers revealed a clear divergence in the kinetics of anti-F and anti-G antibody responses across pediatric and adult age groups. In all pediatric age groups anti-F IgG were more abundant than anti-G, contrasting with adults where no significant differences were found between antigens (Wilcoxon matched-pairs signed-rank test, Figure 9A). In the youngest infants this difference was slighter, reflecting a greater contribution of maternal transfer. As patient age increased, the disparity between antigen titers became more pronounced, indicating a predominance of anti-F IgG as active immunity begins to emerge. Notably, in the 24–72 months old cohort, a marked divergence was observed, with anti-F titer greatly exceeding those against G. Accordingly, the F/G IgG titer ratio in this age group differed significantly from all other age groups (Kruskal–Wallis test with Dunn’s *post hoc* correction; Figure 9B), underscoring a delayed or less robust anti-G antibody response relative to anti-F during the period of active immune maturation (Figure 9B). Representative ELISA titration curves for different age groups can be found in Supplementary Figure 6. These findings collectively suggest that while anti-G antibodies are efficiently transferred, the development of anti-G responses, potentially influenced by the structural and immunomodulatory properties of the G protein’s CCD, lags behind, becoming quantitatively similar to anti-F only after repeated antigenic encounters.

## 4 Discussion

This study provides a comprehensive characterization of the RSV G protein central conserved domain (CCD), elucidating its biochemical properties, immunomodulatory capacity, and displaying its potential impact on humoral immunity in pediatric populations, with direct implications for vaccine design. The principal findings demonstrate that the CCD’s four cysteine residues adopt a predominantly oxidized state consistent with disulfide-stabilized folding a putatively native CX3C chemokine-like motif at low concentrations (Figure 2), which has been previously shown to bind the CX3CR1 receptor expressed on human airway epithelial cells (hEAC) and diverse immune cell subsets (Lee et al., 2018). Functional assays revealed that the CCD, when expressed as a recombinant peptide (Gpep), selectively suppresses activation of dendritic cells (Figure 6), reduces OT-II CD4^+^ T cell proliferation and IFN-*γ* production (Figure 7) and suppresses activation of neutrophils in-vitro, without inducing cytotoxicity (Figure 8; Supplementary Figure 4). To the best of our knowledge, this is the first report showing the effect of RSV G on neutrophils. Neutrophil recruitment is an early event following RSV infection acting as a first line of defense in viral clearance and also promoting adaptive immunity (Li et al., 2019). Treatment with the Gpep significantly attenuated neutrophil inflammatory responses in-vitro, as evidenced by impaired chemotaxis, reduced generation of reactive oxygen species (ROS), diminished release of myeloperoxidase (MPO), and suppression of neutrophil extracellular trap (NET) formation (Figures 8A–E). Thus, collectively our results show that the G peptide is able to modulate both the innate and adaptive immune responses. Our findings, derived from the canonical A2 sequence (UniProt accession number P03423), highlight the need for future quantitative studies on point mutations found in circulating strains to assess their impact on immunosuppressive function. Notably, this immunosuppressive activity is contingent upon the peptide’s oligomerization state and concentration, with monomeric Gpep displaying robust function, while oligomeric forms and/or membrane-bound G (mG) lack immunomodulatory effects. Recent work showed that secreted RSV sG, released from infected cells, acts as a paracrine signaling molecule on nearby uninfected airway epithelial cells (Meineke et al., 2026; Segovia et al., 2012). The interaction of sG with TLR2 initiates MyD88-NF-κB signaling that drives the production of proinflammatory cytokines, ROS accumulation and provides a priming signal for NLRP3 inflammasome. This, in turn, makes the uninfected cells more susceptible to severe inflammatory responses and programmed cell death -pyroptosis- upon subsequent RSV infection. Thus, RSV sG facilitates viral infection through both inflammatory and immunosuppressive mechanisms. It drives excessive inflammation and pyroptosis via TLR2 priming, which aids viral dissemination, while its CX3C motif disrupts chemokine signaling, weakening targeted antiviral cellular immunity.

The versatility of CCD disulfide bonds emerged as a key determinant of structural and functional outcomes. Oxidative folding studies under varied redox conditions demonstrated that Gpep rapidly attained its native state, with minimal accumulation of folding intermediates sensitive to disulfide reshuffling (Figure 3). At higher concentrations, Gpep undergoes intermolecular disulfide bonding (Figure 4), suggesting that oligomerization of membrane-bound G may be similarly mediated. However, the existence and nature of intercatenary disulfide bonds in mG remain unresolved, and detailed structural and redox characterization of membrane-anchored G produced in HAE cells will be required to address this issue (King et al., 2021; Kwilas et al., 2009; Escribano-Romero et al., 2004). Furthermore, there is evidence indicating that G can form oligomers with F in the viral membrane through disulfide-mediated interactions, supporting the notion that cysteine residues within G are redox-active and capable of undergoing isomerization (Arumugham et al., 1989; Low et al., 2008). Local confinement and elevated effective concentration within the lipid bilayer, might facilitate disulfide isomerization and crosslinks between F and G membrane-bound proteins. Molecular dynamics simulations, size-exclusion chromatography, and fluorescence spectroscopy collectively indicate that native, disulfide-bonded Gpep behaves as an extended monomer in solution, likely due to the presence of flexible N-terminal regions, whereas the reduced peptide retains a partially compact, molten globule–like conformation (Figure 5). These findings highlight the concentration-dependent conformational plasticity of the CCD, which may underlie distinct functional profiles in the soluble and membrane-bound forms of the G protein. Future studies incorporating full-length G expressed in eukaryotic systems will be required to directly assess how these features influence folding, oligomerization, and immunomodulatory activity.

Structurally, the distinction between monomeric and oligomeric Gpep species is consequential for immunomodulatory activity. The data show that only the monomeric, soluble Gpep retains the ability to attenuate innate immune activation, suppressing key responses in dendritic cells and neutrophils, while oligomeric Gpep and UV-RSV, where mG predominates, fail to elicit these effects (Supplementary Figure 5). This implicates sG as the principal mediator of RSV-driven immunosuppression via the CX3C motif, with oligomerization and membrane association possibly imposing conformational constraints that may preclude receptor engagement and functional modulation. Reversible formation and reduction of disulfide bonds in redox-sensitive motifs within viral ectodomains can induce structural rearrangements, resulting in functional changes. For instance, redox regulated mechanisms for viral entry and membrane fusion have been described for HIV Gp120/Gp41 protein (Gallina et al., 2002; Barbouche et al., 2003). Allosteric disulfide bridges have also been described in multiple extracellular molecules such as integrins, where the cysteine residues act as redox switches modulating structure–function relationships (Eble, 2025). In the case of RSV CCD, the differential activities observed underscore the importance of CCD structure and presentation in shaping the host immune response. Literature addressing the differential impact of sG and mG on immune responses is based on studies using recombinant viruses (Agac et al., 2023; Bergeron and Tripp, 2021; Anderson et al., 2021). Across these models, the absence of sG is generally associated with enhanced proinflammatory responses, including increased expression of cytokines and adhesion molecules and exacerbated airway inflammation, although the magnitude and nature of these effects remain strongly dependent on cellular context and experimental conditions.

Observations in clinically healthy pediatric cohorts, with no evidence of acute infection at the time of sampling or during the past month, further delineate the functional consequences of CCD immunomodulation on humoral immunity. Analysis of anti-F and anti-G IgG antibody titers in children aged 0–72 months revealed a pronounced bias toward anti-F responses during early exposures, with anti-G titers lagging, particularly in the 24–72-month group (Figure 9). This temporal dissociation was not observed in adults, where antibody levels against both antigens equilibrate, nor in infants within the window of maternal antibody persistence, who display ratios similar to adults (Fuentes et al., 2020; Borochova et al., 2020). The delayed rise of anti-G antibodies in children may reflect the immunosuppressive activity of the CCD. Notably, these findings align with previous reports that associate lower anti-G titers with increased disease severity (Nziza et al., 2024) and highlight the potential for sG-mediated immunosuppression to influence the trajectory of immune maturation and clinical outcomes (Bukreyev et al., 2008; Harcourt et al., 2006). Alternative factors may also contribute to the observed differences in anti-F/anti-G antibody ratios, particularly during early life. The RSV F protein is a well-established immunodominant antigen, characterized by higher epitope density, structural stability, and more efficient processing and presentation, which may intrinsically favor anti-F–biased responses during primary infections. In addition, differences in antigen routing and presentation pathways, as well as the extensive glycosylation of G (Wolfert and Boons, 2013), could limit proteolytic processing and MHC-II presentation of G-derived peptides, further skewing early humoral responses toward F. While these factors likely contribute to the observed humoral bias, they do not fully account for the age-dependent dissociation identified in actively immunized children, nor for its resolution in adulthood. We therefore propose that sG-mediated immunomodulation acts in parallel with intrinsic antigenic properties of F and G, selectively delaying the establishment of robust anti-G responses during early exposures.

Importantly, both RSV A and RSV B lineages co-circulated during the study period (2023–2024) (Perroud et al., 2025). Because our pediatric cohort spans both seasons, individual antibody responses cannot be assigned to a specific viral genotype. Nevertheless, this age-mixed exposure provides an internal control to assess the contribution of antigenic variability to the observed humoral patterns. Notably, anti-F antibody titers—directed against a highly conserved viral antigen—exhibited greater dispersion than anti-G responses, as reflected by broader interquartile ranges across pediatric age groups and supported by variance comparisons using a median-based Levene (Brown–Forsythe) test (*p* < 0.05). Given that the RSV G protein is substantially more variable than F, strain-specific antigenic differences would be expected to preferentially increase variability in anti-G responses. The opposite pattern observed here argues against viral genotype as the primary driver of the age-dependent uncoupling of anti-F and anti-G immunity, supporting instead a dominant role for host developmental and immunomodulatory factors. Whether the secreted sG protein shapes the humoral immune response during early RSV infections remains an open question that requires further research.

Collectively, these results have significant implications for RSV vaccine design, particularly in pediatric populations at heightened risk for severe disease. The capacity of the G protein CCD to suppress PAMP-mediated activation of innate immunity during the early stages of infection, when sG concentrations are highest, may dampen the activation of antigen-presenting cells and skew the development of protective immune memory. Coupled with additional viral mechanisms of immune evasion, such as IFN suppression by NS1 and NS2 proteins (Van Royen et al., 2022; Merritt et al., 2023), RSV employs a multifaceted strategy to silence host defenses. Clinical and experimental evidence indicates that anti-G antibodies are associated with milder disease in infants (Nziza et al., 2024), and display potent neutralizing and protective activity in primary airway models and animal systems (Nziza et al., 2024; Jeong et al., 2015). Therefore, effective vaccine strategies must not only target key antigenic determinants but also counteract CCD-mediated immunosuppression, ensuring robust activation of both innate and adaptive immunity in young children. Elucidating the mechanisms by which the CX3C motif modulates immune responses will be critical for preventing severe RSV disease and establishing durable protective immunity.

## Data Availability

The raw data supporting the conclusions of this article will be made available by the authors, without undue reservation.
